# Disparities in Posthospitalization Disposition Following Tracheotomy: A National Analysis

**DOI:** 10.1002/oto2.70129

**Published:** 2025-05-16

**Authors:** Radhika Duggal, Sarah Benyo, Elizabeth N. Dewey, Rebecca C. Nelson, Paul C. Bryson, Michael S. Benninger, Brandon Hopkins, William S. Tierney

**Affiliations:** ^1^ Cleveland Clinic Lerner College of Medicine Case Western Reserve University Cleveland Ohio USA; ^2^ Head and Neck Institute, Cleveland Clinic Foundation Cleveland Ohio USA; ^3^ Center for Populations Health, Quantitative Health Sciences, Cleveland Clinic Cleveland Ohio USA

**Keywords:** discharge disposition, disparities, social determinants of health, tracheostomy, tracheotomy

## Abstract

**Objective:**

Previous studies have demonstrated the impact of sociodemographic factors on disease development, management, and outcomes in adult and pediatric populations. Given that postoperative management is key in reducing complications following a tracheotomy, we assessed the impact of sociodemographic factors on a patient's discharge disposition.

**Study Design:**

Cross‐sectional study.

**Setting:**

Health Care Utilization Project's (HCUP) National Inpatient Survey (NIS).

**Methods:**

The HCUP NIS was queried for all patients undergoing tracheotomy between 2017 and 2021. All analyses were performed using R Version 4.3.1 survey procedures to account for strata and cluster effects.

**Results:**

We identified 81,069 admissions during which a tracheotomy was performed and, after appropriate weighting for the HCUP NIS survey design, found that 15.1% of admissions resulted in routine discharge, 4.5% transferred to a short‐term hospital, 52.3% transferred to a skilled nursing facility (SNF)/intermediate care facility (ICF)/other facility, 16.9% discharged with home health care. Admissions routinely discharged had the lowest median (interquartile range) age (48 [23, 61] years), whereas admissions resulting in death or transfer to a SNF/ICF/other facility type had the greatest age (63 [53, 70] years). On both univariable and multivariable analyses, age, race, sex, insurance type, geographic region, and hospital size were associated with discharge disposition.

**Conclusion:**

Our study highlights that disparities exist among patient populations and were found in both unadjusted and adjusted analyses. Further attention and resource allocation for the care of patients with a tracheostomy may work toward identifying sources of disparity, which may be modified to improve patient care.

Tracheotomy in adults is most commonly performed in patients who have had difficulty weaning off a ventilator or who have suffered trauma or a neurologic insult.^1^ It is also performed in the context of infectious or neoplastic processes, particularly those involving the upper airway. The use of tracheostomies has profound implications on cost, resource utilization, and patient outcomes during acute hospitalization and after discharge. These outcomes are also influenced by patient sociodemographic characteristics.^2^, ^3^, ^4^ Patients of lower socioeconomic status are more likely to suffer from chronic diseases,^5^ and many studies have shown that uninsured patients or those with Medicaid have disproportionately worse health outcomes than patients with private insurance or Medicare.^6^ Furthermore, it has been found in a variety of disease processes that discharge disposition is associated with overall patient outcomes, including cost of care, 30‐day readmission, and mortality.^7^, ^8^, ^9^ Previous literature similarly indicates that discharge disposition is influenced by hospital characteristics, including hospital type and region, independent of illness severity.^10^ Given the complex needs of patients with a tracheostomy after discharge, it is important to identify factors that are associated with discharge disposition.

Demographic and socioeconomic factors have been shown to impact tracheostomy patients specifically. In ventilator‐dependent adult patients, racial and ethnic minorities have higher odds of receiving a tracheostomy compared with white patients.^11^ Females and non‐English‐speaking patients have been shown to undergo tracheotomy significantly later after initial intubation as compared to English‐speaking patients and men.^12^ Much of the existing literature focuses on demographic features in the pediatric tracheostomy population. A study of the incidence of pediatric tracheostomies in the United States showed that children of black race were more likely to undergo tracheotomy and to have increased hospital length of stay, even after adjusting for potential confounders.^13^ Additionally, demographic factors including age, community type, race, and hospital factors such as hospital type and geographic region have been shown to influence discharge disposition in pediatric tracheostomy patients.^3^ These studies have provided a groundwork to examine associations of demographic factors with discharge disposition in the adult tracheostomy population.

As individuals with tracheostomies have a variety of functional, physical, and socioeconomic challenges from the initial tracheotomy procedure through hospital discharge and caring for the tracheostomy at home,^14^, ^15^ postoperative education and evaluation of comorbidities, safety, and potential barriers to adequate care after discharge are of utmost importance for determining discharge disposition.^15^, ^16^ Given that postoperative management is key in reducing complications following a tracheostomy,^17^ we sought to assess the impact of sociodemographic factors on a patient's discharge disposition. We hypothesized that sociodemographic factors would significantly impact discharge disposition.

## Methods

The Health Care Utilization Project's (HCUP) National Inpatient Survey (NIS) is the largest publicly available all‐payer inpatient care database in the United States and contains data pertaining to more than 7 million hospital stays. The HCUP NIS was queried for all tracheostomy‐dependent adults between 2017 and 2021 utilizing relevant International Statistical Classification of Diseases, Tenth Revision (ICD‐10) procedure codes (detailed in Supplemental Table S1, available online). This study utilized publicly available data from the HCUP NIS and did not require approval by our institutional review board.

Discharge weights unique to each year's data set were utilized, and all analyses were performed using R Version 4.3.1 survey procedures to account for strata and cluster effects. For comparisons of continuous and categorical variables by discharge disposition, the Wilcoxon rank‐sum test for complex survey samples and Rao‐Scott chi‐square tests were utilized, respectively. The top 20 admissions diagnoses and procedures coded for this cohort were identified (Supplemental Tables S2‐S4, available online). All ICD‐10 code definitions utilized in the study are enumerated in Supplemental Table S5, available online. Ten clinically relevant admissions diagnoses and procedures were included in further analysis. A “supported discharge” is defined as any discharge requiring support greater than routine discharge to home with self‐care, including discharge home with home health care (HHC), discharge to a skilled nursing facility (SNF)/intermediate care facility (ICF)/other facility type, and transfer to another hospital.

To investigate the impact of patient and hospital characteristics on discharge disposition, adjusted odds ratios are reported from multivariable logistic regression models. Multivariable analyses included variables that have previously been suggested to contribute to discharge disposition including age, length of hospitalization, race, sex, income, patient location, payer, hospital bed size, hospital ownership, hospital location, hospital region, top admissions diagnoses, and top procedures as covariates. All variables, including definitions of categorical variables, are defined per the HCUP data element descriptions. All analyses were completed with routine discharge as the reference. For all analyses beyond initial description of the cohort, admissions discharged against medical advice (AMA), admissions resulting in death during hospitalization, or admissions for which the discharge disposition is not known were excluded.

## Results

In total, 34,486,914 admissions between 2017 and 2021 were screened for inclusion, and 81,069 admissions met criteria. Of all discharges, most (n. unweighted: 42,392) were transfers to other facilities (such as SNFs, ICFs, long‐term acute care facilities, etc.). Discharge to home with HHC contributed 13,699 discharges, and routine discharges with self‐care contributed 12,205 discharges.

### Patient Characteristics

Results from univariable analyses of patient characteristics are found in Table 1. Admissions discharged home (15.1%) were disproportionately younger (median [interquartile range] 42.3 [23, 61]), Hispanic (19%), male (16%), self‐pay (36%), and lived in micropolitan (defined as a county with a population between 10,000 and 50,000, per the HCUP data element definitions) or noncore area (17%) (*P* < .001). Admissions transferred to a short‐term hospital (4.5% of discharges) were disproportionately of Hispanic (6%) or other (including Asian, Pacific Islander, Native American, or admissions designated as other, 6%) race, in the highest quartile of income (5%), and had an expected primary payer of no charge/other (8%) or Medicaid (5%) (*P* < .001). Admissions transferred to SNF/ICF/other facility types (52.3%) were disproportionately older (median 62 [51, 72]), black (56%), female (55%), lowest income quartile (53%), Medicare (61%), and lived in a large metropolitan area (55%) (*P* < .001). Admissions discharged home with HHC (16.9%) were disproportionately white (19%), male (18%), highest income quartile (19%), private insurance (19%), and lived in small and medium micropolitan areas (19%) (*P* < .001).

**Table 1:** Descriptive Statistics for Demographic Characteristics From Admissions That Included Tracheostomy in Health Care Utilization Project National Inpatient Survey Data Between 2017 and 2021^a^

	Routine discharge	Transfer to short‐term hospital	Transfer SNF/ICF/other facility	Discharge with home health care	Discharge against medical advice	Death	
Demographic characteristic	N = 12,205, wtd%: 15.1 (14.6, 15.6)	N = 3667 wtd%: 4.5 (4.3, 4.7)	N = 42,392 wtd%: 52.3 (51.7, 52.9)	N = 13,699 wtd%: 16.9 (16.4, 17.4)	N = 312 wtd%: 0.4 (0.3‐0.4)	N = 8790 wtd%: 10.8 (10.6‐11.1)	*P* value
Age	48 [23, 61]	56 [34, 67]	62 [51, 72]	59 [47, 67]	52 [38, 60]	63 [53, 72]	**<.001**
Length of stay	10 [6, 28]	24 [13, 42]	26 [16, 40]	11 [7, 23]	10 [5, 25]	29 [18, 46]	**<.001**
Race							**<.001**
White	14.3% (13.8, 14.9)	4.1% (3.9, 4.3)	52.2% (51.5, 52.9)	18.8% (18.2, 19.4)	0.4% (0.3, 0.4)	10.2% (9.9, 10.5)	
Black	14.5% (13.7, 15.3)	4.3% (4.0, 4.8)	56.0% (55.0, 57.0)	14.0% (13.3, 14.6)	0.5% (0.4, 0.6)	10.7% (10.2, 11.2)	
Hispanic	18.5% (17.5, 19.6)	5.6% (5.1, 6.1)	47.4% (46.2, 48.6)	15.5% (14.6, 16.5)	0.3% (0.2, 0.4)	12.7% (12.0, 13.5)	
Other	15.9% (14.7, 17.2)	5.8% (5.3, 6.4)	51.0% (49.6, 52.5)	14.6% (13.7, 15.5)	0.3% (0.2, 0.4)	12.3% (11.6, 13.0)	
Sex							**<.001**
Male	16.1% (15.6, 16.7)	4.7% (4.5, 5.0)	50.5% (49.8, 51.1)	17.5% (17.0, 18.0)	0.4% (0.4, 0.5)	10.7% (10.4, 11.0)	
Female	13.4% (12.8, 14.0)	4.2% (3.9, 4.5)	55.1% (54.4, 55.9)	16.0% (15.4, 16.5)	0.3% (0.2, 0.3)	11.0% (10.7, 11.4)	
Income quartile from ZIP code							**<.001**
First quartile	15.3% (14.7, 16.0)	4.2% (4.0, 4.6)	53.3% (52.5, 54.1)	15.7% (15.1, 16.3)	0.5% (0.4, 0.6)	10.9% (10.5, 11.3)	
Second quartile	15.2% (14.5, 15.8)	4.4% (4.1, 4.8)	52.5% (51.6, 53.4)	16.9% (16.3, 17.6)	0.3% (0.3, 0.4)	10.6% (10.2, 11.1)	
Third quartile	15.1% (14.4, 15.9)	4.4% (4.1, 4.7)	51.5% (50.6, 52.4)	17.9% (17.2, 18.7)	0.3% (0.2, 0.4)	10.8% (10.3, 11.2)	
Fourth quartile	13.7% (12.9, 14.5)	5.0% (4.6, 5.5)	51.5% (50.4, 52.5)	18.5% (17.6, 19.4)	0.2% (0.2, 0.3)	11.0% (10.5, 11.6)	
Patient location (NCHS urban‐rural code)							**<.001**
Large metropolitan	13.7% (13.2, 14.3)	4.4% (4.1, 4.6)	54.4% (53.6, 55.1)	16.1% (15.6, 16.7)	0.4% (0.3, 0.4)	11.0% (10.7, 11.4)	
Small and medium metropolitan	16.3% (15.6, 17.0)	4.9% (4.5, 5.3)	49.5% (48.5, 50.4)	18.5% (17.9, 19.2)	0.4% (0.3, 0.5)	10.4% (10.0, 10.8)	
Micropolitan and noncore	17.3% (16.3, 18.4)	4.2% (3.8, 4.6)	50.2% (49.1, 51.4)	17.2% (16.3, 18.1)	0.3% (0.2, 0.4)	10.8% (10.2, 11.4)	
Expected primary payer							**<.001**
Medicare	7.6% (7.3, 8.0)	3.7% (3.4, 4.0)	60.7% (60.0, 61.4)	15.8% (15.2, 16.3)	0.2% (0.2, 0.3)	12.0% (11.6, 12.4)	
Medicaid	22.3% (21.5, 23.2)	5.2% (4.8, 5.6)	45.4% (44.5, 46.3)	16.9% (16.3, 17.6)	0.7% (0.6, 0.9)	9.5% (9.0, 9.9)	
Private insurance	17.1% (16.3, 17.9)	4.8% (4.5, 5.2)	48.5% (47.6, 49.5)	19.4% (18.6, 20.2)	0.2% (0.2, 0.3)	10.0% (9.5, 10.4)	
Self pay	36.1% (33.8, 38.4)	4.5% (3.8, 5.5)	30.5% (28.5, 32.7)	15.4% (13.9, 17.1)	0.8% (0.5, 1.3)	12.6% (11.3, 14.0)	
No charge or other	22.4% (20.7, 24.1)	7.9% (6.9, 9.0)	43.2% (41.2, 45.2)	15.1% (13.8, 16.6)	0.6% (0.4, 0.9)	10.9% (9.8, 12.1)	

Bold *P* values denote statistically significant.

Abbreviations: ICF, intermediate care facility; NCHS, National Center for Health Statistics; SNF, skilled nursing facility; wtd, weighted.

^a^ Numeric data are presented as weighted median [interquartile range]. Categorical data are presented as the weighted proportion (95% confidence interval).

### Hospital Characteristics

Results from univariable analyses of hospital characteristics are found in Table 2. Admissions discharged home (15.1% of all) were disproportionately from hospitals with a large bed size (16%), public ownership (22%), urban teaching status (16%), and located in the south (18%) (*P* < .001). Admissions discharged to a short‐term hospital (4.5%) were disproportionately from small (6%) and medium (5%) bed size hospitals, rural (8%), and located in the west (7%) (*P* < .001). Admissions discharged to a SNF/ICF/other facility type (52.3%) were disproportionately from small (55%) and medium (56%) bed size, private (54%), urban nonteaching (59%), and Midwest (57%) hospitals (*P* < .001). Finally, admissions discharged home with HHC (16.9%) were disproportionately from large (19%), public (18%), urban teaching (18%) or rural (17%), northeast (18%) or south (17%) hospitals (*P* < .001).

**Table 2:** Descriptive Statistics for Hospital Characteristics From Admissions That Included Tracheostomy in Health Care Utilization Project National Inpatient Survey Data Between 2017 and 2021^a^

	Routine discharge	Transfer to short‐term hospital	Transfer to SNF/ICF/other facility type	Discharge with home health care	Discharge against medical advice	Death	
Hospital characteristic	N = 12,205, wtd%: 15.1 (14.6, 15.6)	N = 3667 wtd%: 4.5 (4.3, 4.7)	N = 42,392 wtd%: 52.3 (51.7, 52.9)	N = 13,699 wtd%: 16.9 (16.4, 17.4)	N = 312 wtd%: 0.4 (0.3‐0.4)	N = 8790 wtd%: 10.8 (10.6‐11.1)	*P* value
Bed size							**<.001**
Small	13.6% (12.5, 14.7)	5.8% (5.3, 6.3)	54.5% (53.0, 55.9)	14.2% (13.1, 15.3)	0.4% (0.3, 0.6)	11.6% (10.9, 12.3)	
Medium	14.0% (13.1, 14.9)	5.2% (4.8, 5.7)	55.9% (54.7, 57.0)	12.8% (12.1, 13.5)	0.4% (0.3, 0.5)	11.7% (11.2, 12.3)	
Large	15.8% (15.1, 16.5)	4.0% (3.7, 4.3)	50.5% (49.7, 51.3)	19.1% (18.4, 19.7)	0.4% (0.3, 0.4)	10.3% (10.0, 10.7)	
Control/ownership							**<.001**
Government nonfederal (public)	21.6% (20.1, 23.2)	4.8% (4.1, 5.5)	45.2% (43.7, 46.7)	17.6% (16.3, 18.9)	0.4% (0.3, 0.5)	10.4% (9.8, 11.1)	
Private	13.8% (13.3, 14.3)	4.5% (4.3, 4.7)	53.7% (53.0, 54.3)	16.8% (16.3, 17.3)	0.4% (0.3, 0.4)	10.9% (10.6, 11.2)	
Location/teaching status							**<.001**
Rural	9.7% (8.5, 11.1)	8.0% (6.9, 9.3)	50.7% (48.5, 53.0)	16.7% (14.8, 18.7)	0.6% (0.3, 1.0)	14.3% (12.9, 15.9)	
Urban nonteaching	9.3% (8.5, 10.1)	5.9% (5.4, 6.5)	58.7% (57.5, 59.9)	12.7% (11.9, 13.5)	0.5% (0.4, 0.7)	12.9% (12.2, 13.7)	
Urban teaching	16.0% (15.4, 16.5)	4.2% (4.0, 4.5)	51.5% (50.8, 52.2)	17.5% (16.9, 18.0)	0.4% (0.3, 0.4)	10.5% (10.2, 10.8)	
Region (US Census)							**<.001**
Northeast	9.8% (9.0, 10.7)	4.6% (4.1, 5.1)	54.9% (53.6, 56.2)	17.8% (16.7, 18.9)	0.3% (0.3, 0.4)	12.6% (11.9, 13.3)	
Midwest	14.1% (13.1, 15.2)	3.3% (3.0, 3.6)	57.1% (55.7, 58.5)	16.5% (15.5, 17.5)	0.3% (0.2, 0.4)	8.7% (8.2, 9.1)	
South	17.9% (17.0, 18.7)	4.2% (3.9, 4.6)	49.3% (48.4, 50.3)	17.3% (16.6, 18.1)	0.4% (0.3, 0.5)	10.8% (10.4, 11.3)	
West	15.7% (14.8, 16.7)	6.7% (6.2, 7.3)	49.9% (48.7, 51.1)	15.5% (14.5, 16.5)	0.5% (0.4, 0.7)	11.7% (11.1, 12.3)	

Bold *P* values denote statistically significant.

Abbreviations: ICF, intermediate care facility; SNF, skilled nursing facility; wtd, weighted.

^a^ Data are presented as the weighted proportion (95% CI).

### Frequent Admissions Diagnoses and Procedures

Table 3 demonstrates 10 frequent admissions diagnoses and procedures present in the cohort stratified by discharge disposition. Admissions with a diagnosis of sepsis, COVID‐19, respiratory failure, cerebral hemorrhage, aspiration, myocardial infarction, and chronic kidney disease were disproportionately likely to be transferred to an SNF/ICF/other facility type. Conversely, admissions with cancer of the head and neck or laryngeal stenosis were disproportionately less likely to be transferred to an SNF/ICF/other facility type, instead having an increased proportion of routine discharges and discharges with HHC. With the exception of bronchoscopy and laryngoscopy, all top procedures resulted in a disproportionate increase in the likelihood of transfer to an SNF/ICF/other facility type.

**Table 3:** Comparison of Most Frequent Admissions Diagnoses and Procedures by Discharge Disposition Following Tracheostomy in Health Care Utilization Project National Inpatient Survey 2017 to 2022^a^

	Routine discharge	Transfer to short‐term hospital	Transfer to SNF/ICF/other facility type	Discharge with home health care	
Admissions diagnosis/procedure	N = 12,205, wtd%: 17.0 (16.4, 17.5)	N = 3667 wtd%: 5.1 (4.9, 5.3)	N = 42,392 wtd%: 58.9 (58.2, 59.6)	N = 13,699 wtd%: 19.0 (18.5, 19.6)	*P* value
Most frequent admissions diagnoses					
Sepsis	7.6% (7.1%, 8.1%)	5.1% (4.7%, 5.6%)	76.5% (75.7%, 77.3%)	10.8% (10.2%, 11.4%)	**<.001**
COVID‐19	5.6% (4.7%, 6.7%)	7.7% (6.5%, 9.1%)	79.3% (77.3%, 81.1%)	7.5% (6.4%, 8.7%	**<.001**
Respiratory failure	15.6% (14.5%, 16.7%)	6.6% (6.0%, 7.4%)	60.9% (59.5%, 62.3%)	16.9% (16.0%, 18.0%)	**<.001**
Tracheostomy‐related complication	31.0% (29.2%, 32.9%)	3.8% (3.1%, 4.6%)	40.3% (38.3%, 42.3%)	24.9% (23.2%, 26.7%)	**<.001**
Cancer of the head or neck	26.5% (25.0%, 28.2%)	1.8% (1.5%, 2.1%)	24.0% (22.8%, 25.2%)	47.7% (46.1%, 49.3%)	**<.001**
Cerebral hemorrhage	7.7% (7.0%, 8.5%)	5.5% (4.9%, 6.2%)	82.7% (81.6%, 83.8%)	4.0% (3.5%, 4.6%)	**<.001**
Aspiration	7.6% (7.1%, 8.1%)	5.1% (4.7%, 5.6%)	76.5% (75.7%, 77.3%)	10.8% (10.2%, 11.4%)	**<.001**
Laryngeal stenosis	38.7% (36.0%, 41.6%)	1.7% (1.2%, 2.6%)	18.4% (16.3%, 20.6%)	41.2% (38.5%, 43.9%)	**<.001**
Myocardial infarction	5.2% (3.9%, 6.8%)	6.0% (4.5%, 7.8%)	80.9% (78.2%, 83.3%)	8.0% (6.4%, 10.0%)	**<.001**
Chronic kidney disease	7.8% (5.8%, 10.4%)	4.5% (3.0%, 6.7%)	76.0% (72.1%, 79.5%)	11.7% (9.2%, 14.7%)	**<.001**
Most frequent procedures during admission					
Respiratory ventilation	11.3% (10.9%, 11.8%)	6.0% (5.7%, 6.4%)	71.4% (70.7%, 72.0%)	11.3% (10.9%, 11.6%)	**<.001**
Insertion of endotracheal airway	8.6% (8.2%, 9.0%)	6.2% (5.8%, 6.6%)	77.2% (76.5%, 77.9%)	8.0% (7.6%, 8.4%)	**<.001**
Insertion of infusion device into superior vena cava	8.5% (8.1%, 9.0%)	6.0% (5.7%, 6.4%)	75.5% (74.8%, 76.2%)	10.0% (9.5%, 10.4%)	**<.001**
Insertion of feeding device into stomach	9.6% (9.1%, 10.1%)	5.0% (4.7%, 5.3%)	72.3% (71.5%, 73.0%)	13.1% (12.6%, 13.6%)	**<.001**
Inspection of tracheobronchial tree, via natural or artificial opening endoscopic	21.3% (20.1%, 22.5%)	5.7% (5.2%, 6.3%)	55.8% (54.5%, 57.2%)	17.2% (16.3%, 18.1%)	**<.001**
Introduction of nutritional substance into upper GI, via natural or artificial opening	12.1% (11.3%, 13.0%)	4.7% (4.3%, 5.2%)	66.1% (64.8%, 67.3%)	17.1% (16.2%, 18.0%)	**<.001**
Performance of urinary filtration	5.8% (5.2%, 6.5%)	6.9% (6.2%, 7.7%)	79.8% (78.6%, 80.9%)	7.5% (6.8%, 8.3%)	**<.001**
Drainage of right pleural cavity, any approach	9.2% (8.4%, 10.2%)	7.1% (6.4%, 7.9%)	74.0% (72.7%, 75.3%)	9.6% (8.8%, 10.5%)	**<.001**
Drainage of right lower lung lobe, any approach	9.0% (8.0%, 10.1%)	6.0% (5.2%, 6.9%)	75.2% (73.6%, 76.7%)	9.8% (8.8%, 10.8%)	**<.001**
Inspection of larynx, via natural or artificial opening	31.4% (29.8%, 32.9%)	4.8% (4.3%, 5.4%)	33.6% (32.2%, 34.9%)	30.2% (28.9%, 31.6%)	**<.001**

Bold *P* values denote statistically significant.

Abbreviations: GI, gastrointestinal; ICF, intermediate care facility; SNF, skilled nursing facility; wtd, weighted.

^a^ Current Procedural Terminology codes utilized in the identification of each diagnosis and procedure are found in Supplemental Table S5, available online. All diagnoses are assessed by presence as the admissions diagnosis, and all procedures are assessed as presence during any time of the admission. Discharges against medical advice and admissions resulting in death are excluded from analysis. Data are presented as the weighted proportion (95% CI).

### Multivariable Regression Analyses: Risk Factors for Supported Discharge

**Figure 1:**
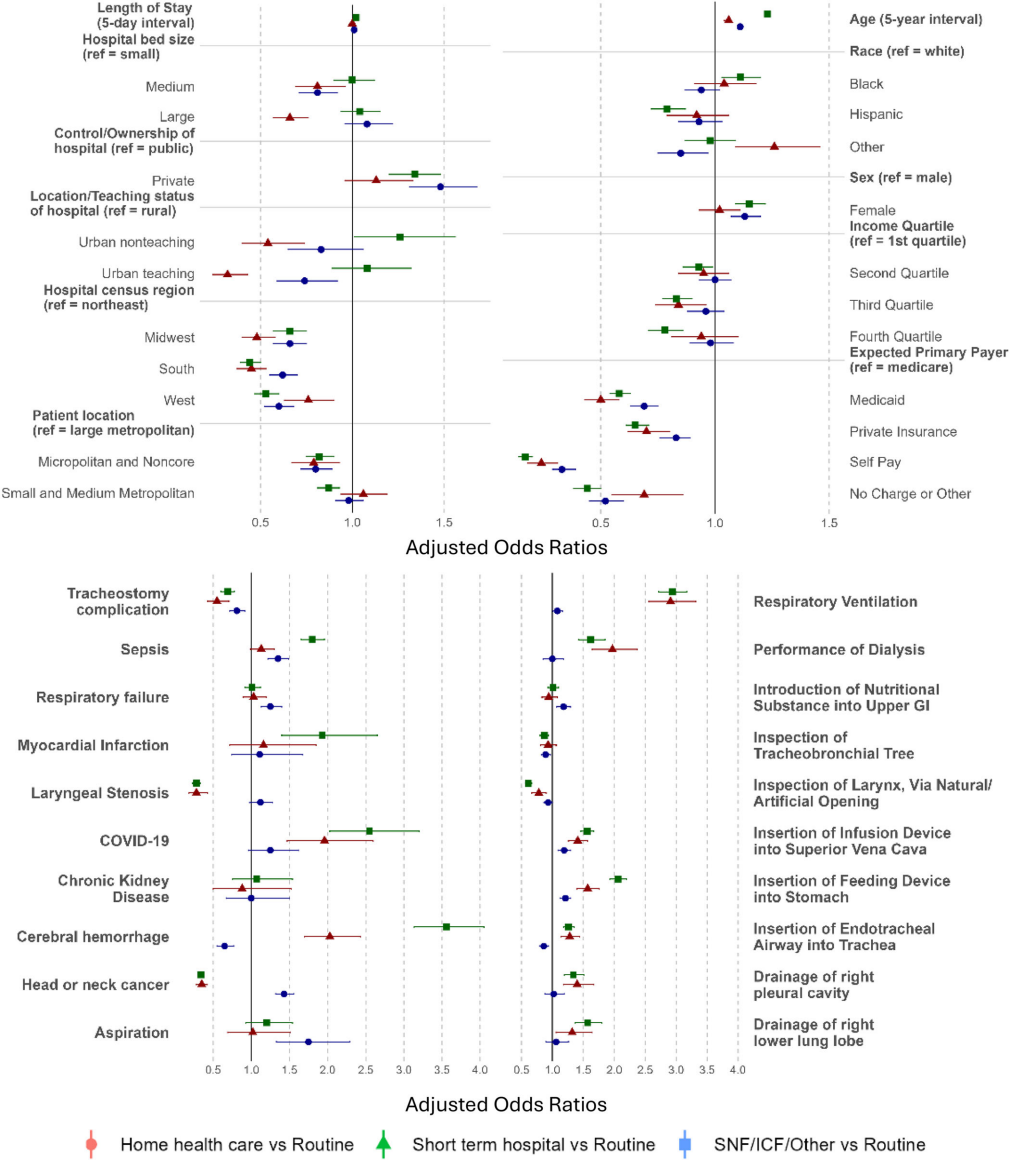
Adjusted odds of (A) patient/hospital characteristics and (B) top admissions diagnoses/procedures. Outcomes relative to discharge home with self‐care: transfer to home health care (blue circle), skilled nursing facility (SNF)/intermediate care facility (ICF)/other facility (green square) or short‐term facility (red triangle). GI, gastrointestinal.

Weighted odds ratios with 95% confidence intervals are reported in Table 4 and Figure 1. Greater odds for discharge to HHC was associated with increasing age (*P* < .001), white race (*P* = .01), female sex (*P* < .001), location in large metropolitan area (*P* < .001), Medicare (*P* < .001), small hospital bed size (*P* = .002), private hospital ownership (*P* < .001), rural status (*P* = .01), and location in the Northeast (*P* < .001). There were greater odds of transfer to a short‐term facility associated with increasing age (*P* < .001), other race (*P* = .001), lowest income quartile (*P* = .009), location in a large metropolitan area (*P* = .004), Medicare (*P* < .001), small hospital bed size (*P* = .02), rural status (*P* < .001), and location in the Northeast (*P* = .002). Finally, there were greater odds of transfer to a SNF/ICF/other facility type with increasing age (*P* < .001), black race (*P* = .007), female sex (*P* < .001), lowest income quartile (*P* = .03), location in a large metropolitan area (*P* < .001), Medicare (*P* < .001), private hospital ownership (*P* < .001), urban nonteaching status (*P* = .04), and location in the Northeast (*P* < .001).

**Table 4:** Adjusted Odds Ratios (aORs) for Discharge Disposition Compared to Routine Discharge Following Admissions for Tracheostomy in Health Care Utilization Project National Inpatient Survey 2017 to 2021

	Discharge with HHC versus routine		Transfer to short‐term hospital versus routine		Transfer to SNF/ICF/other facility versus routine	
Characteristic	aOR (95% conf. interval)	*P* value	aOR (95% conf. interval)	*P* value	aOR (95% conf. interval)	*P* value
Intercept	0.61 (0.44, 0.85)	**.003**	0.66 (0.41, 1.05)	.08	0.23 (0.17, 0.32)	**<.001**
Age (5‐y interval)	1.11 (1.10, 1.12)	**<.001**	1.06 (1.04, 1.07)	**<.001**	1.23 (1.22, 1.24)	**<.001**
LOS (5‐d increase)	1.01 (1.01, 1.02)	**<.001**	1.0 (1.0, 1.01)	.13	1.02 (1.01, 1.02)	**<.001**
Race						
White	(Reference)		(Reference)		(Reference)	
Black	0.94 (0.87, 1.02)	.16	1.04 (0.91, 1.18)	.58	1.11 (1.03, 1.20)	**.007**
Hispanic	0.93 (0.84, 1.03)	.15	0.92 (0.79, 1.06)	.25	0.79 (0.72, 0.87)	**<.001**
Other	0.85 (0.75, 0.97)	**.01**	1.26 (1.09, 1.46)	**.001**	0.98 (0.87, 1.09)	.68
Sex						
Male	(Reference)		(Reference)		(Reference)	
Female	1.13 (1.07, 1.20)	**<.001**	1.02 (0.93, 1.11)	.73	1.15 (1.09, 1.22)	**<.001**
Income quartile from ZIP code						
First quartile	(Reference)		(Reference)		(Reference)	
Second quartile	1.00 (0.93, 1.07)	.99	0.95 (0.84, 1.06)	.35	0.93 (0.86, 0.99)	**.03**
Third quartile	0.96 (0.88, 1.04)	.32	0.84 (0.74, 0.96)	**.009**	0.83 (0.77, 0.90)	**<.001**
Fourth quartile	0.98 (0.89, 1.08)	.69	0.94 (0.81, 1.10)	.45	0.78 (0.71, 0.86)	**<.001**
Patient location (NCHS urban‐rural code)						
Large metropolitan	(Reference)		(Reference)		(Reference)	
Small and medium metropolitan	0.98 (0.91, 1.06)	.63	1.06 (0.94, 1.19)	.38	0.87 (0.81, 0.93)	**<.001**
Micropolitan and noncore	0.80 (0.72, 0.89)	**<.001**	0.79 (0.67, 0.93)	**.004**	0.82 (0.75, 0.90)	**<.001**
Expected primary payer						
Medicare	(Reference)		(Reference)		(Reference)	
Medicaid	0.69 (0.63, 0.75)	**<.001**	0.50 (0.43, 0.58)	**<.001**	0.58 (0.54, 0.63)	**<.001**
Private insurance	0.83 (0.76, 0.89)	**<.001**	0.70 (0.62, 0.80)	**<.001**	0.65 (0.61, 0.71)	**<.001**
Self pay	0.33 (0.29, 0.39)	**<.001**	0.24 (0.18, 0.31)	**<.001**	0.17 (0.14, 0.20)	**<.001**
No charge or other	0.52 (0.45, 0.60)	**<.001**	0.69 (0.55, 0.86)	**<.001**	0.44 (0.38, 0.50)	**<.001**
Hospital bed size						
Small	(Reference)		(Reference)		(Reference)	
Medium	0.81 (0.71, 0.92)	**.002**	0.81 (0.69, 0.96)	**.02**	1.00 (0.90, 1.12)	.95
Large	1.08 (0.96, 1.22)	.19	0.66 (0.57, 0.76)	**<.001**	1.04 (0.94, 1.15)	.46
Control/ownership of hospital						
Government nonfederal (public)	(Reference)		(Reference)		(Reference)	
Private	1.48 (1.31, 1.68)	**<.001**	1.13 (0.96, 1.33)	.13	1.34 (1.20, 1.48)	**<.001**
Location/teaching status of hospital						
Rural	(Reference)		(Reference)		(Reference)	
Urban nonteaching	0.83 (0.65, 1.06)	.13	0.54 (0.40, 0.74)	**<.001**	1.26 (1.01, 1.56)	**.04**
Urban teaching	0.74 (0.59, 0.92)	**.01**	0.32 (0.24, 0.43)	**<.001**	1.08 (0.89, 1.32)	.42
Hospital census region						
Northeast	(Reference)		(Reference)		(Reference)	
Midwest	0.66 (0.57, 0.75)	**<.001**	0.48 (0.40, 0.58)	**<.001**	0.66 (0.57, 0.75)	**<.001**
South	0.62 (0.55, 0.70)	**<.001**	0.45 (0.37, 0.53)	**<.001**	0.44 (0.39, 0.50)	**<.001**
West	0.60 (0.52, 0.68)	**<.001**	0.76 (0.63, 0.90)	**.002**	0.53 (0.47, 0.60)	**<.001**
Sepsis (yes/no)	1.35 (1.22, 1.49)	**<.001**	1.13 (0.99, 1.30)	.07	1.80 (1.65, 1.96)	**<.001**
COVID‐19 (yes/no)	1.25 (0.96, 1.62)	.10	1.96 (1.47, 2.60)	**<.001**	2.55 (2.03, 3.20)	**<.001**
Respiratory failure (yes/no)	1.25 (1.13, 1.40)	**<.001**	1.03 (0.89, 1.20)	.68	1.01 (0.92, 1.12)	.82
Tracheostomy‐related complication (yes/no)	0.81 (0.72, 0.92)	**<.001**	0.55 (0.43, 0.71)	**<.001**	0.69 (0.60, 0.78)	**<.001**
Cancer of the head or neck (yes/no)	1.43 (1.32, 1.56)	**<.001**	0.35 (0.28, 0.42)	**<.001**	0.34 (0.31, 0.37)	**<.001**
Cerebral hemorrhage (yes/no)	0.65 (0.55, 0.77)	**<.001**	2.03 (1.70, 2.43)	**<.001**	3.56 (3.13, 4.05)	**<.001**
Aspiration (yes/no)	1.75 (1.33, 2.29)	**<.001**	1.02 (0.69, 1.51)	.92	1.20 (0.93, 1.54)	.16
Laryngeal stenosis (yes/no)	1.12 (0.98, 1.28)	.10	0.28 (0.18, 0.43)	**<.001**	0.28 (0.23, 0.33)	**<.001**
Myocardial infarction (yes/no)	1.11 (0.74, 1.67)	.61	1.16 (0.72, 1.85)	.54	1.93 (1.40, 2.65)	**<.001**
Chronic kidney disease (yes/no)	1.00 (0.67, 1.50)	1.00	0.88 (0.50, 1.52)	.64	1.07 (0.75, 1.54)	.70
Respiratory ventilation (yes/no)	1.08 (1.01, 1.17)	**.03**	2.91 (2.56, 3.32)	**<.001**	2.94 (2.72, 3.17)	**<.001**
Insertion of endotracheal airway into trachea (yes/no)	0.86 (0.79, 0.94)	**<.001**	1.28 (1.14, 1.44)	**<.001**	1.26 (1.18, 1.35)	**<.001**
Insertion of infusion device into superior vena cava (yes/no)	1.19 (1.09, 1.29)	**<.001**	1.41 (1.26, 1.57)	**<.001**	1.56 (1.46, 1.67)	**<.001**
Insertion of feeding device into stomach (yes/no)	1.21 (1.12, 1.29)	**<.001**	1.57 (1.40, 1.75)	**<.001**	2.06 (1.93, 2.20)	**<.001**
Inspection of tracheobronchial tree, via natural or artificial opening endoscopic (yes/no)	0.89 (0.82, 0.97)	**.006**	0.93 (0.81, 1.06)	.26	0.87 (0.80, 0.94)	**<.001**
Introduction of nutritional substance into upper GI, via natural or artificial opening (yes/no)	1.18 (1.07, 1.29)	**<.001**	0.94 (0.83, 1.08)	.40	1.01 (0.93, 1.10)	.81
Performance of dialysis (yes/no)	1.00 (0.85, 1.18)	.97	1.97 (1.64, 2.37)	**<.001**	1.62 (1.42, 1.85)	**<.001**
Inspection of larynx, via natural or artificial opening (yes/no)	0.93 (0.86, 1.00)	.07	0.78 (0.67, 0.90)	**<.001**	0.61 (0.56, 0.67)	**<.001**
Drainage of right pleural cavity, any approach (yes/no)	1.02 (0.88, 1.19)	.75	1.40 (1.18, 1.67)	**<.001**	1.34 (1.19, 1.51)	**<.001**
Drainage of right lower lung lobe, any approach (yes/no)	1.06 (0.89, 1.26)	.54	1.32 (1.06, 1.64)	**.01**	1.57 (1.37, 1.80)	**<.001**

Bold *P* values denote statistically significant.

Abbreviations: GI, gastrointestinal; HHC, home health care; ICF, intermediate care facility; LOS, length of stay; NCHS, National Center for Health Statistics; SNF, skilled nursing facility.

Admissions diagnoses of sepsis (*P* < .001), COVID‐19 (*P* < .001, respiratory failure (*P* < .001), cerebral hemorrhage (*P* < .001), aspiration (*P* < .001), and myocardial infarction (*P* < .001) were associated with a significantly increased odds of a supported discharge. Procedures associated with increased odds of a supported discharge included respiratory ventilation (*P* < .001), insertion of an endotracheal airway into the trachea (*P* < .001), insertion of an infusion device into the superior vena cava (*P* < .001), insertion of a feeding device into the stomach (*P* < .001), introduction of nutritional substances into the upper gastrointestinal (*P* < .001), performance of dialysis (*P* < .001), drainage of the right pleural cavity (*P* < .001), and drainage of the right lower lung lobe (*P* < .001).

## Discussion

Postoperative management of tracheostomy patients, including assessment of the patient's and family's ability to care for a tracheostomy at home, overall safety, and potential barriers to care, is a crucial component for determining discharge disposition. To the authors' knowledge, this is the largest database study to date evaluating the relationship between social determinants of health and discharge disposition after tracheotomy. In a study of national trends in tracheostomy for mechanically ventilated patients from 1993 to 2012, there was a notable shift toward more frequent discharges to long‐term care facilities (40%‐72%) and fewer discharges to home (21%‐13%).^11^ Of the discharges included in our study (2017‐2021), most patients (59%) were discharged to an SNF/ICF/other facility, which may similarly reflect this nationwide shift in discharge disposition.

In a multivariable regression comparing discharges to HHC, short‐term hospitals, and SNF/ICF/other facilities to routine discharge home with self‐care, there were significant differences in all patient and hospital characteristics assessed in the present study. When evaluating discharge disposition by age, older patients were more likely to be transferred to an SNF/ICF/other facility or pass away before discharge, whereas younger patients were more likely to be discharged home. Additionally, patients with Medicare (age 65 and older) were more likely to have a supported discharge. These findings may reflect that, with increasing age, there are more comorbidities that may restrict a patient's ability to care for themselves at home and may therefore impact discharge disposition.^18^ This may be attributable to the etiology of the tracheostomy requirement necessitating an increased level of care in an older population.

Patients in the lowest income quartile had increased odds of discharge to a short‐term hospital or SNF/ICF/other facility as compared to routine discharge home. Income is a surrogate marker of socioeconomic status, and patients in lower income quartiles have been associated with higher odds of hospital readmissions, increased hospital length of stay, increased hospital charges, greater rate of tracheotomy, and shorter overall life expectancy, as compared with those in higher income quartiles.^11^, ^19^, ^20^, ^21^, ^22^ Multiple studies have shown that comorbidities are highest in patients of lower income quartiles, and thus tend to have a greater need for health care services,^21^, ^23^, ^24^ which may contribute to more frequent discharges to facilities in this subpopulation. Our results may reflect that the indications for tracheotomy required a higher level of care after discharge as well as decreased financial resources to care for oneself after tracheostomy placement among lower‐income patients.

When compared to routine discharge home, white patients were more likely to be discharged home with HHC but less likely to be discharged to short‐term hospitals or SNF/ICF/other facility than patients of all other races (Table 3). This reinforces racial trends observed for discharge disposition following tracheotomy in the pediatric setting and after other surgical procedures. For example, a large regional database study of patients undergoing total knee replacement found that African American patients are more likely to be discharged to SNF or inpatient rehabilitation facilities as compared to white patients, and it has previously been found that patients discharged to SNF or rehabilitation facilities had higher odds of 90‐day hospital readmission.^25^ Therefore, the present study adds important information to the literature regarding racial disparities in discharge disposition, which may serve as a surrogate for outcomes such as complications and readmissions. It is important to consider that race may serve as a proxy for other factors that may be driving the difference in discharge disposition. Multiple previous studies have demonstrated racial differences in socioeconomic status, which may contribute to disparities in health.^26^, ^27^ Supplemental Table S6, available online, demonstrates the intersection between race and income quartile and reinforces that, though race is a significant predictor of discharge disposition even in our adjusted, multivariable analysis, there is a greater percentage of non‐white individuals in lower income brackets, which may be contributing to the observed nationwide trends. Whether race may be serving as a proxy for other factors, such as differences in resource availability, patient comfort caring for tracheostomies, communication barriers, or other factors, is not addressed in this research. The specific association between discharge disposition and outcomes for patients with a tracheostomy requires further investigation.

Patients in large metropolitan areas were more likely to be discharged to SNF/ICF/other facilities, and hospital location in the Northeast was associated with greater odds of transfer to SNF/ICF/other facilities or short‐term hospital. This regional trend is similar to findings of discharge disposition in the pediatric tracheostomy population.^3^ Previous studies have demonstrated that discharge disposition to facilities is dependent on the geographic location of the hospital and nearby facilities.^10^, ^28^ Larger urban centers may have greater facility availability or higher existing bases of postcare facilities, which may contribute to our findings.

Urban nonteaching hospitals were associated with increased odds of discharging to SNF/ICF/other. Studies have demonstrated an absolute mortality benefit of medical and surgical patients receiving treatment at teaching hospitals as compared to nonteaching hospitals.^29^, ^30^ Tracheotomies are more likely to be performed in urban, teaching hospitals,^11^ and teaching hospitals are often larger than nonteaching hospitals, invest in cutting‐edge technology, and have workforce characteristics generally associated with better quality of care for these medically complex patients.^31^ There is likely greater resource availability in teaching hospitals including residents, fellows, nurse coordinators, and advanced practice providers, which may allow for more focused patient education and discharge coordination,^32^ in turn increasing discharges to home.

Medicaid and self‐pay admissions were more likely to be discharged home compared to patients with other insurance types. Medicaid tends to have lower reimbursement rates for long‐term acute care facilities compared with Medicare and private insurance, and admitted Medicaid patients are less likely to transfer to such facilities after discharge.^33^, ^34^ As such, patients' means to pay for a facility or to cover costs not funded by their insurance would ultimately limit access to the full range of long‐term and short‐term medical care available after hospital discharge. Although routine discharge to home is often considered the goal for discharge, particularly in quality assessment metrics, it is important to recognize that it is unlikely that the increased odds of discharge home associated with Medicaid and self‐pay admissions represent a positive quality trend. Instead, it may be worthwhile to reevaluate the goals of discharge disposition, particularly for hospitalizations that may require a greater level of postdischarge support.

Hospital size and ownership were also noted to be associated with discharge disposition, with greater odds of patients discharging to short‐term hospitals or SNF/ICF/other from small and privately funded hospitals. In terms of hospital ownership, there are generally three types of ownership including public, private not‐for‐profit (PNFP), and private for‐profit (PFP). Previous studies have focused on the effects of hospital ownership on quality of care and patient outcomes. A systematic review of the performance of private and public hospitals across Europe suggested that public hospitals are at least as efficient or more efficient than private hospitals, and the growth of the private sector of hospitals is not related to improvements in performance or quality of care.^35^ Another systematic review and meta‐analysis of outcomes by hospital ownership demonstrated that mortality rates were higher in PFP facilities compared to public and PNFP hospitals.^36^ It is possible that hospitals provide differing incentives that contribute to differences in patient care. Previous studies have suggested that hospital performance assessments and ranking mechanisms may bias discharge dispositions to favor positive quality metrics.^37^, ^38^ Similarly, increased hospital length of stay increases costs for health care systems. In smaller private systems, there may be fewer resources to coordinate discharge needs and facilitate patient education, resulting in a higher level of discharge disposition.

Overall, findings of this study indicate significant variability in the discharge disposition of patients with a tracheostomy associated with social determinants of health after adjusting for patient characteristics, including admissions diagnoses and procedures, and hospital characteristics. Standardization of tracheostomy care within a hospital has been found to reduce mortality, Intensive Care Unit length of stay, and ventilator dependence, while improving quality of life metrics.^39^ In addition to standardization of care, it is important that efforts are made to bridge the observed sociodemographic disparities and improve access to care for patients with a tracheostomy. An interprofessional care team including discharge planning support, such as that provided by a social worker, may improve consistency of care for patients with a tracheostomy and better equip patients with resources needed for optimal discharge, ultimately improving outcomes in this patient group.^3^, ^40^ Future studies regarding the impact of these interprofessional care teams on patient outcomes and discharge dispositions, and their interaction with patient sociodemographic factors, are needed.

Strengths of this study include the large sample size from a national database. The main limitation of this study is the use of administrative data that rely on ICD‐10 coding, which lacks details regarding disease severity, comorbidities, and other factors that may contribute to disposition after tracheotomy. Although we are able to estimate disease severity and comorbidities present through ICD‐10 codes, we acknowledge that billing codes are imprecise measures of true patient experiences. It is unfortunately not feasible to definitively identify whether this results in an overestimation or underestimation of true disease severity and comorbidity status. Finally, although our analysis adjusts for the diagnosis of COVID‐19, it is important to recognize that the COVID‐19 pandemic may provide potential for skewed outcomes that cannot be completely accounted for. Future prospective, electronic medical record‐based studies may allow for detailed analyses of discharge disposition, including the impact of variables that are not included in the HCUP NIS database.

## Conclusions

The present study demonstrates that hospital discharge disposition after tracheotomy in an adult population is associated with various patient‐ and hospital‐related factors after adjusting for the impact of heterogenous admissions diagnoses and procedures among the cohort. In a multivariable analysis, we found that factors associated with an increased odds of discharge to a short‐term facility or home with HHC relative to routine discharge included increasing age, white race, female sex, the lowest income quartile, and hospital location. Factors associated with a greater odds of transfer to an SNF/ICD/other facility type included increasing age, black race, female sex, the lowest income quartile, and hospital location. These findings suggest multifactorial disparities contributing to differences in discharge dispositions for patients undergoing tracheotomy. Future efforts to identify and implement strategies to minimize the impact of these disparities on patient care are needed.

## Author Contributions

**Radhika Duggal**, conceptualization, data acquisition, analysis, and interpretation, manuscript drafting and reviewing; **Sarah Benyo**, data analysis and interpretation, manuscript drafting and reviewing; **Elizabeth N. Dewey**, data acquisition and analysis, manuscript review; **Rebecca C. Nelson**, data interpretation, manuscript review; **Paul C. Bryson**, data interpretation, manuscript review; **Michael S. Benninger**, data interpretation, manuscript review; **Brandon Hopkins**, conceptualization, data analysis and interpretation, manuscript review; **William S. Tierney**, conceptualization, data analysis and interpretation, manuscript review.

## Disclosures

### Competing interests

We have no conflicts of interest to disclose.

### Funding source

None.

## Supporting information

Supporting information.
