# Supplementary material for: Disparities in Posthospitalization Disposition Following Tracheotomy: A National Analysis
Source: OTO Open. 2025 May 16;9(2):e70129. doi: 10.1002/oto2.70129 (PMC12082083; doi:10.1002/oto2.70129)
Supplement: Supplementary file 1 — Supporting information. [file OTO2-9-e70129-s001.docx]

**Supplementary Tables.**

**Supplementary Table 1.** ICD-10 Codes utilized to identify patients with a tracheostomy.

| **ICD-10 PCS** | **Definition** | **N unwtd.** |
| --- | --- | --- |
| 0B21XFZ | Change Tracheostomy Device in Trachea, External Approach | 28569 |
| 0BP10FZ | Removal of Tracheostomy Device from Trachea, Open Approach | 29 |
| 0BP13FZ | Removal of Tracheostomy Device from Trachea, Perc Approach | 18 |
| 0BP14FZ | Removal of Trach Dev from Trachea, Perc Endo Approach | 8 |
| 0BP17FZ | Removal of Tracheostomy Device from Trachea, Via Opening | 54 |
| 0BP18FZ | Removal of Tracheostomy Device from Trachea, Endoscopic | 66 |
| 0BP1XFZ | Removal of Tracheostomy Device from Trachea, External Approach | 3147 |
| 0B110F4 | Bypass Trachea to Cutaneous with Tracheostomy Dev, Open Approach | 61542 |
| 0B110Z4 | Bypass Trachea to Cutaneous, Open Approach | 344 |

**Supplementary Table 2.** Top 20 admission diagnoses

| **ICD-10 Dx** | **Definition** | **N unwtd.** |
| --- | --- | --- |
| A419 | Sepsis, unspecified organism | 9254 |
| U071 | COVID-19 | 3185 |
| J9621 | Acute and chronic respiratory failure with hypoxia | 2629 |
| A4189 | Other specified sepsis | 2507 |
| J9601 | Acute respiratory failure with hypoxia | 1907 |
| J9503 | Malfunction of tracheostomy stoma | 1145 |
| C329 | Malignant neoplasm of larynx, unspecified | 1092 |
| C321 | Malignant neoplasm of the supraglottis | 1028 |
| J9622 | Acute and chronic respiratory failure with hypercapnia | 1006 |
| S065X9A | Traumatic subdural hemorrhage with loss of consciousness for an unspecified amount of time during an initial encounter | 998 |
| J690 | Pneumonitis due to inhalation of food and vomit | 833 |
| C01 | Malignant neoplasm of base of tongue | 821 |
| S066X9A | Traumatic subarachnoid hemorrhage with loss of consciousness of unspecified duration, initial encounter | 805 |
| C029 | Unspecified malignant neoplasm of the tongue | 778 |
| C411 | Malignant neoplasm of mandible | 667 |
| A4102 | Sepsis caused by Methicillin resistant Staphylococcus aureus | 644 |
| J386 | Stenosis of larynx | 584 |
| I214 | Non-ST elevation (NSTEMI) myocardial infarction | 583 |
| Z430 | Encounter for attention to tracheostomy | 574 |
| J9509 | Other tracheostomy complication | 531 |

**Supplementary Table 3.** Top 20 diagnoses any time during admission, including admission diagnosis

| **ICD-10 Dx** | **Definition** | **N unwtd.** |
| --- | --- | --- |
| J9601 | Acute respiratory failure with hypoxia | 26973 |
| I10 | Essential (primary) hypertension. | 23706 |
| E870 | Hyperosmolality and hypernatraemia | 20959 |
| A419 | Sepsis, unspecified organism | 19340 |
| N179 | Acute kidney failure that is unspecified | 19169 |
| D62 | Acute posthemorrhagic anemia | 18628 |
| R6521 | Severe sepsis with septic shock | 18447 |
| E872 | Acidosis | 18253 |
| E785 | Hyperlipidemia, unspecified | 17063 |
| J690 | Pneumonitis due to inhalation of food and vomit | 16685 |
| J9621 | Acute and chronic respiratory failure with hypoxia | 16192 |
| E871 | Hypo-osmolality and hyponatremia | 15556 |
| Z9911 | Dependence on respirator [ventilator] status. | 14848 |
| E876 | Hypokalemia | 14505 |
| R1310 | Dysphagia, Unspecified | 14320 |
| Z87891 | Nicotine dependence | 12367 |
| K219 | Gastro-oesophageal reflux disease without oesophagitis | 12268 |
| N390 | Urinary tract infection, site not specified | 11608 |
| N170 | Acute kidney failure with tubular necrosis | 11426 |
| E43 | Unspecified severe protein-energy malnutrition | 10803 |

**Supplementary Table 4.** Top 20 procedures during admission

| **ICD-10 PCS** | **Definition** | **N unwtd.** |
| --- | --- | --- |
| 5A1955Z | Respiratory Ventilation, Greater than 96 Consecutive Hours | 55716 |
| 02HV33Z | Insertion of Infusion Device into Superior Vena Cava, Percutaneous Approach | 35585 |
| 0BH17EZ | Insertion of Endotracheal Airway into Trachea | 35538 |
| 0DH63UZ | Insertion of Feeding Device into Stomach, Percutaneous Approach | 27608 |
| 0BJ08ZZ | Inspection of Tracheobronchial Tree, Via Natural or Artificial Opening Endoscopic | 15940 |
| 3E0G76Z | Introduction of Nutritional Substance into Upper GI, Via Natural or Artificial Opening | 15410 |
| 30233N1 | Transfusion of Nonautologous Red Blood Cells into Peripheral Vein, Percutaneous Approach | 13778 |
| 03HY32Z | Insertion of Monitoring Device into Upper Artery, Percutaneous Approach | 12157 |
| 5A1D70Z | Performance of Urinary Dialysis, <6 hrs/day | 10884 |
| 5A1945Z | Respiratory Ventilation, 24-96 Consecutive Hours | 10368 |
| 0BH18EZ | Insertion of Endotracheal Airway into Trachea, Via Natural or Artificial Opening Endoscopic | 9391 |
| 0CJS8ZZ | Inspection of Larynx, Via Natural or Artificial Opening Endoscopic | 9122 |
| B548ZZA | Ultrasonography of Superior Vena Cava, Guidance | 6368 |
| 0DJ08ZZ | Inspection of Upper Intestinal Tract, Via Natural or Artificial Opening Endoscopic | 6002 |
| 4A133B1 | Monitoring of Arterial Pressure, Peripheral, Percutaneous Approach | 5942 |
| 0W9930Z | Drainage of Right Pleural Cavity with Drainage Device, Percutaneous Approach | 5058 |
| 5A12012 | Performance of Cardiac Output, Single, Manual | 4458 |
| 4A133J1 | Monitoring of Arterial Pulse, Peripheral, Percutaneous Approach | 4341 |
| 0B9F8ZX | Drainage of Right Lower Lung Lobe, Via Natural or Artificial Opening Endoscopic, Diagnostic | 4333 |
| 3E033XZ | Introduction of Vasopressor into Peripheral Vein, Percutaneous Approach | 4225 |

**Supplementary Table 5.** ICD-10 code definitions utilized in the study

| Diagnosis/Procedure | ICD-10 codes included |
| --- | --- |
| Sepsis | A40, A41, R65.2 |
| COVID-19 | U07.1 |
| Respiratory failure | J96 |
| Tracheostomy-related complication | J95 |
| Cancer of the head or neck | C00-C14, C30-33, C73, |
| Cerebral hemorrhage | I60-I62, S06.4X - 506.6x |
| Aspiration pneumonitis | J69 |
| Laryngeal Stenosis | J38 |
| Myocardial Infarction | I21 |
| Chronic Kidney Disease | N18, I12, I13 |
| Respiratory Ventilation greater than 96 consecutive hours, 24-96 consecutive hours, or less than 24 consecutive hours | 5A1935Z, 5A1945Z, 5A1955Z, 5A19054 |
| Insertion of Endotracheal Airway into Trachea | 0BH17EZ, 0BH17DZ |
| Insertion of Infusion Device into Superior Vena Cava | 02HV33Z, 02HV43Z, 02HV03Z |
| Insertion of Feeding Device into Stomach | 0DH63UZ, 0DH60UZ, 0DH64UZ, 0DH67UZ, 0DH68UZ |
| Inspection of Tracheobronchial Tree, Via Natural or Artificial Opening Endoscopic | 0BJ07ZZ , 0BJ08ZZ |
| Introduction of Nutritional Substance into Upper GI, Via Natural or Artificial Opening | 3E0G76Z, 3E0G36Z, 3E0G86Z |
| Performance of Urinary Dialysis, <6 hrs/day, 6-18hrs/day, >18 hrs/day | 5A1D60Z, 5A1D70Z, 5A1D80Z, 5A1D90Z |
| Inspection of Larynx, Via Natural or Artificial Opening | 0CJS7ZZ, 0CJS8ZZ |
| Drainage of right pleural cavity, any approach | 0W9900, 0W9900Z, 0W990ZX, 0W990ZZ, 0W9930, 0W9930Z, 0W993ZX, 0W993ZZ, 0W9940, 0W9940Z, 0W994ZX, 0W994ZZ |
| Drainage of right lower lung lobe, any approach | 0B9F00, 0B9F00Z, 0B9F0ZX, 0B9F0ZZ, 0B9F30, 0B9F30Z, 0B9F3ZX, 0B9F3ZZ, 0B9F40, 0B9F40Z, 0B9F4ZX, 0B9F4ZZ, 0B9F70, 0B9F70Z, 0B9F7ZX, 0B9F7ZZ, 0B9F80, 0B9F80Z, 0B9F8ZX, 0B9F8ZZ |

**Supplementary Table 6.** Comparison of income quartiles by race among the full cohort of patients undergoing tracheostomy in HCUP NIS 2017-2022.

|  | | **Income Quartile** | | | |
| --- | --- | --- | --- | --- | --- |
|  |  | **First Quartile** | **Second Quartile** | **Third Quartile** | **Fourth Quartile** |
| **Race** | **While** | **27.6% (26.8%, 28.3%)** | **28.1% (27.5%, 28.6%)** | **24.4% (23.9%, 25.0%)** | **19.9% (19.2%, 20.7%)** |
|  | **Black** | **53.3% (52.1%, 54.5%)** | **21.3% (20.5%, 22.1%)** | **15.5% (14.8%, 16.2%)** | **9.9% (9.3%, 10.6%)** |
|  | **Hispanic** | **38.8% (37.4%, 40.2%)** | **25.5% (24.5%, 26.6%)** | **23.0% (22.0%, 24.0%)** | **12.7% (11.9%, 13.6%)** |
|  | **Other** | **27.8% (26.4%, 29.2%)** | **23.0% (22.0%, 24.0%)** | **23.6% (22.6%, 24.7%)** | **25.6% (24.4%, 26.9%)** |
| *Note:* Data is presented as the weighted proportion (95% CI). | | | | | |
